# The prevalence of cardiometabolic multimorbidity and its association with physical activity, diet, and stress in Canada: evidence from a population-based cross-sectional study

**DOI:** 10.1186/s12889-019-7682-4

**Published:** 2019-10-24

**Authors:** Brodie M. Sakakibara, Adebimpe O. Obembe, Janice J. Eng

**Affiliations:** 1 Department of Physical Therapy, Faculty of Medicine, University of British Columbia, Vancouver, BC V6T 1Z3 Canada; 2 Faculty of Health Sciences, Simon Fraser University, Burnaby, Canada; 3 Rehabilitation Research Program, Vancouver Coastal Health Research Institute, Vancouver, BC V5Z 2G9 Canada

## Abstract

**Background:**

Cardiometabolic multimorbidity (CM) is defined as having a diagnosis of at least two of stroke, heart disease, or diabetes, and is an emerging health concern, but the prevalence of CM at a population level in Canada is unknown. The objectives of this study were to quantify the: 1) prevalence of CM in Canada; and 2) association between CM and lifestyle behaviours (e.g., physical activity, consumption of fruits and vegetables, and stress).

**Methods:**

Using data from the 2016 Canadian Community Health Survey, we estimated the overall and group prevalence of CM in individuals aged ≥50 years (*n* = 13,226,748). Multiple logistic regression was used to quantify the association between CM and lifestyle behaviours compared to a group without cardiometabolic conditions.

**Results:**

The overall prevalence of CM was 3.5% (467,749 individuals). Twenty-two percent (398,755) of people with diabetes reported having another cardiometabolic condition and thus CM, while the same was true for 32.2% (415,686) of people with heart disease and 48.4% (174,754) of stroke survivors. 71.2% of the sample reported eating fewer than five servings of fruits and vegetables per day. The odds of individuals with CM reporting zero minutes of physical activity was 2.35 [95% CI = 1.87 to 2.95] and having high stress was 1.89 [95% CI = 1.49 to 2.41] times the odds of the no cardiometabolic condition reference group. The odds of individuals with all three cardiometabolic conditions reporting zero minutes of physical activity was 4.31 [95% CI = 2.21 to 8.38] and having high stress was 3.93 [95% CI = 2.03 to 7.61].

**Conclusion:**

The number of Canadians with CM or at risk of CM is high and these individuals have lifestyle behaviours that are associated with adverse health outcomes. Lifestyle behaviours tend to diminish with increasing onset of cardiometabolic conditions. Lifestyle modification interventions focusing on physical activity and stress management for the prevention and management CM are warranted.

## Background

Healthcare management of patients who have multiple chronic diseases, or multimorbidity, is traditionally based on disease-specific strategies that are independent of one another. This approach often leads to fragmented and episodic care, resulting in healthcare inefficiencies and increased patient burden to navigate multiple providers and systems [1]. Moreover, most patients with multimorbidity develop complications that are clinically complex and present as unique healthcare challenges. These complexities are often poorly understood, which means these patients have unmet health care needs. In fact, existing research shows significant associations between multimorbidity and adverse health outcomes, including higher rates of premature mortality [2], increased healthcare use [3], more complications of treatment beyond the effects of individual conditions [1], limitations with physical function and greater disability [4], lower quality of life [5, 6], and psychological issues, such as depression and distress [7, 8].

While research clearly identifies multimorbidity as a preeminent health issue, much of the findings are limited to non-specific forms of multimorbidity, with little emphasis on particular chronic disease clusters [9]. Without research on the prevention, treatment, and management of diseases that we know cluster together — such as stroke, heart disease and diabetes — health professionals and policy-makers will remain with limited guidance or evidence as to how to approach care and health promotion decisions. Moreover, the issues and challenges associated with multimorbidity will continue and further burden our health system as well as public and population health, and health promotion initiatives.

Stroke, heart disease, and diabetes are three of the most prevalent chronic diseases worldwide with substantial social and economic burden. They are cardiometabolic diseases with similar etiologies (e.g., lifestyle behaviours) that are leading causes of health resource use, hospitalizations, morbidity and mortality in Canada [10, 11]. Together, they account for more than 70,000 deaths per in Canada [12] and $34 billion annually in direct and indirect costs [10, 13]. While much evidence exists on the epidemiology, prevention and management of each individual cardiometabolic disease on their own [11, 14–25], there remains little research on the impact of the diseases in combination (i.e., cardiometabolic multimorbidity), despite their high risk of co-occurrence.

Cardiometabolic multimorbidity (CM) is defined as having a diagnosis of at least two of stroke, heart disease, or diabetes, and is emerging as a key health concern given recent evidence that it is associated with an exponentially increased risk of mortality [2]. In a study of 689,300 individuals, the sex-adjusted mortality rate for individuals without any cardiometabolic condition at 60 years of age was 6.8 per 1000 person-years [2]. By contrast, the age- and sex-adjusted rates were 16.1 among those with a history of stroke, 16.8 with heart disease, and 15.6 with diabetes, between 32.0 and 32.8 for those with two of the conditions, and 59.5 per 1000 person-years for those with all three conditions [2]. Furthermore, another recent study of over 120,000 adults has shown strong associations between higher body mass index and increased risk of CM [26]. After adjustment for sociodemographic and lifestyle factors, compared with individuals with a healthy weight, the risk of developing CM in overweight individuals was 2.0 [95% CI 1.7 to 2.4] times higher, 4.5 [95% CI 3.5 to 5.8] times higher for individuals with class I obesity, and 14.5 [95% CI 10.1 to 21.0] times higher for individuals with classes II and III obesity combined [26].

Lifestyle behaviour modification is the primary non-pharmacologic strategy for the management and prevention of cardiometabolic risk [27]. Physical activity several times a week, combined with a healthier dietary intake has been shown to alleviate risk and complications [27], as has stress lowering measures. Healthy lifestyles are also protective against premature mortality [28, 29] which may have important implications for people with CM. For example, due to poorer functional status commonly associated with multimorbidity [30], individuals with increasing numbers of cardiometabolic conditions experience difficulties managing their lifestyle, resulting in inactivity, poor nutrition, and high stress, and thus higher rates of premature mortality. Furthermore, lifestyle behaviours are also well known modifiable risk factors for each of stroke, heart disease, and diabetes [31–33]. A vast majority of individuals, however, are engaging in high risk lifestyles. For example, among Canadians: 85% of adults are not active enough to meet physical activity guidelines [34]; 69% spend the majority of their time in sedentary activities [34]; and 50% consume fewer servings of fruits and vegetables than that recommended to experience health benefits [35]. Inactivity, poor diet, and less than optimal amounts of stress combined with an aging population are resulting in increasing numbers of Canadians with cardiometabolic conditions, and thus an increasing risk of CM.

While previous studies have examined risk factors for CM, and how CM influences mortality, no previous study has examined the association between CM, physical activity, diet, and stress. In this study, our objectives are to quantify: 1) the prevalence of CM; and 2) the association between individual cardiometabolic conditions and CM on physical activity, diet and stress among Canadians 50 years of age or older. We anticipate that the findings in this paper will lead to more tailored or individualized treatment depending on the severity and numbers of cardiometabolic conditions.

## Methods

The reporting of the methods and results in this cross-sectional study follows the Statistical Analyses and Methods in the Published Literature Guidelines [36].

### Data source

The Canadian Community Health Survey (CCHS) is a national survey that collects information related to health status among Canadian residents aged ≥12 years [37]. Approximately 65,000 individuals residing in all areas of Canada are randomly sampled every year. The following groups are excluded: full-time members of the Canadian Forces, Canadians living on reserves/Aboriginal settlements, in long-term care institutions or in the Quebec health regions of Région du Nunavik and Région des Terres-Cries-de-la-Baie-James (representing < 3% of the Canadian population aged 12 and over). The survey takes approximately 45 min to complete and participation is voluntary. We used data from the 2016 CCHS from respondents aged ≥50 years, so as to include data from the entire baby-boom generation (i.e., born between 1946 and 1964, ages 52–72 in 2016) that is largely responsible for driving health system priorities and reform [38].

### Variables

We included sociodemographic and health status information, such as: age, sex (male/female), body mass index (BMI), marital status (married/common-law vs other), household income (i.e., total income received by all household members, from all sources, before taxes and deductions in the past 12 months) (<$20,000, $20,000 to $49,999, $50,000 to $79,999, ≥$80,000), and education (less than high-school, high-school graduate, post-secondary education). We also included data on BMI because higher BMI has been shown to increase the risk of CM [26]. To determine a diagnosis of stroke, heart disease, or diabetes, respondents were asked by CCHS researchers: “We’re interested in conditions diagnosed by a health professional and that are expected to last or have already lasted 6 months or more. Do you: suffer from the effects of stroke?; have heart disease?; have diabetes?

#### Group (key independent variable of interest)

We stratified respondents into eight mutually exclusive diagnostic groups, similar to previous CM studies [2]: 1) no cardiometabolic condition; 2) stroke; 3) heart disease; 4) diabetes; 5) stroke and heart disease; 6) stroke and diabetes; 7) heart disease and diabetes; and 8) stroke, heart disease, and diabetes.

#### Lifestyle behaviours (dependent variables)

We examined three lifestyle risk factors common to each of stroke, heart disease, and diabetes [31].

- *Physical activity* was assessed by self-reported minutes of moderate to vigorous activity (i.e., sweat a little and breather harder) per week. For our analyses, we dichotomized the ordinal categories into ‘at least moderately active’ (physically active at or below Canadian Physical Activity Guidelines (i.e., 150 min of moderate to vigorous activity per week) [39] vs. ‘inactive’ (no physical activity minutes reported). We used no physical activity minutes reported as the cut point between categories to investigate the extreme nature CM might have reduced activity.
- *Diet* was assessed using the Daily Consumption of Fruits and Vegetables variable. For our analyses, we dichotomized the ordinal categories into usually ‘less than 5 servings/day’ vs. usually ‘5+ servings/day’. We used 5 as the cut point given its proximity to the recommended number of servings (i.e., 7 servings) in Canada’s Food Guide [40], and because the next category asks about 10 servings/day.
- *Stress* was assessed using the Perceived Life Stress variable, which we dichotomized into ‘low stress’ (most days were ‘not at all stressful’, ‘not very stressful’ or ‘a bit stressful’) vs. “high stress (most days were ‘quite a bit stressful’ or ‘extremely stressful’.

### Analyses

Descriptive statistics were used to characterize the respondents with continuous variables presented as means and standard deviations (SD) and categorical variables as percentages and frequency counts.

#### Objective 1

Overall and group prevalence of CM were calculated from the number of respondents reporting a history of at least two of stroke, heart disease, or diabetes relative to the total number of respondents.

#### Objective 2

Multivariable binary logistic regression was used to quantify the association between number of cardiometabolic conditions and lifestyle behaviors of physical activity, diet, and stress. After adjusting for age and sex we estimated the odds of being physically inactive, eating less fruits and vegetables, and having more stress for each group using the ‘no cardiometabolic condition’ as the reference. We adjusted for age because stroke, heart disease, and diabetes are aging related disease, and for sex given the unique aspects of cardiovascular health in women and sex differences as they relate to prevention, diagnosis, and management of cardiometabolic diseases [41, 42].

To account for survey design effects such as clustering and unequal selection probabilities, and to ensure that the results were representative of the Canadian population, we applied Statistics Canada’s calibrated design and bootstrap weights to obtain 2016 population level point estimates and their 95% confidence intervals.

All data were analyzed using WesVar version 5.1 at a significance level of *p* < 0.05.

## Results

### Objective 1 – overall and group prevalence

The weighted sample of 2016 CCHS respondents translated to a population of 13,226,748 community-living Canadians aged ≥50 years. 13.5% (~ 1.78 million) had a history of diabetes, 9.8% (~ 1.29 million) had heart disease, and 2.3% (299,437) were living with effects from a stroke. Twenty-two percent (398,755) of people with diabetes reported having another cardiometabolic condition and thus CM, while the same was true for 32.2% (415,686) of people with heart disease and 48.4% (174,754) of stroke survivors. Individuals with no cardiometabolic condition reported the highest household incomes and education levels, whereas individuals with stroke reported the lowest household incomes and education levels. Body mass index (BMI) increased with increasing numbers of cardiometabolic condition, from 26.5 in people with no condition, to 28.2 in people with one condition, to 29.1 in people with CM. Individuals with diabetes reported higher body mass indices than those with stroke or heart disease.

Overall, 3.5% (467,749) of Canadians aged 50 years and older reported having CM, with slightly more than half (51.8%) being female. The combination of heart disease and diabetes was the most prevalent form of CM (2.2%, 292,995 people), followed by stroke and heart disease (0.5% 68,994 people), stroke, heart disease, and diabetes (0.4%, 53,697 people), and then stroke and diabetes (0.4%, 52,063 people). Fifty percent of people with CM reported no physical activity minutes, per week, 73% reported eating fewer then 5 servings of fruits and vegetables per day, and 22% reported having higher stress. Table 1 presents the sample characteristics by mutually exclusive diagnostic groups.

**Table 1:** Sample characteristics by mutually exclusive diagnostic groups (*n* = 13,226,748)

Characteristics	No cardiometabolic condition	Stroke	Heart disease	Diabetes	Stroke & Heart disease	Stroke & Diabetes	Heart disease & Diabetes	Stroke, Heart disease & Diabetes
Prevalence:								
%	78.4	0.9	6.6	10.5	0.5	0.4	2.2	0.4
(n)	(10,374,278)	(124,683)	(877,672)	(1,382,368)	(68,994)	(52,063)	(292,995)	(53,697)
Age (mean, SD):	62.9, 9.6	70.8, 10.7	69.0, 11.2	66.0, 9.4	74.9, 10.7	70.8, 9.4	69.3, 9.8	68.6, 8.9
Male sex								
%	46.7	46.1	56.9	51.2	50.2	52.3	58.1	63.7
n	4,844,787	57,479	499,395	707,772	34,635	27,229	170,230	34,204
Body mass index (mean, SD)	26.5, 5.1	26.0, 4.9	26.9, 5.2	29.1, 5.8	25.7, 6.2	29.4, 6.4	29.4, 6.4	30.3, 7.2
Married/Common Law								
(%)	69.8	59.9	66.0	66.5	56.5	48.2	56.1	72.8
(n)	7,241,246	74,685	579,264	919,275	38,982	25,094	164,370	39,091
Household income (%, n C$):								
< 20,000	6.0, 622,457	10.3, 12,842	8.7, 76,357	8.6, 118,883	14.0, 9659	20.3, 10,569	19.5, 57,134	11.8, 6336
20,000-49,999	23.2, 2,406,832	41.7, 51,993	32.1, 281,733	32.1, 443,740	37.8, 26,080	48.8, 25,407	33.2, 97,274	41.3, 22,177
50,000-79,999	21.0, 2,178,598	23.5, 29,301	24.0, 210,641	22.3, 308,268	18.0, 12,429	9.9, 5154	16.0, 46,879	18.2, 9773
≥ 80,000	49.8, 5,166,390	24.5, 30,547	35.2, 308,941	37.0, 511,476	30.2, 20,836	21.0, 10,933	31.3, 91,707	28.7, 15,411
Highest education level (%, n):								
Less than highschool	16.4, 1,701,382	29.9, 37,579	225,562	362,180	35.5, 24,493	22,699	37.0, 108,408	43.4, 23,304
Highschool graduate	23.3, 2,417,207	25.6, 31,919	20.3, 178,167	24.7, 341,445	31.0, 21,388	16.7, 8695	20.4, 59,771	21.4, 11,491
Post-secondary education	60.3, 6,255,689	44.5, 55,484	54.1, 474,821	49.1, 678,743	33.5, 23,113	39.7, 20,669	42.7, 125,109	35.2, 18,901

*C$* Canadian dollars

### Objective 2 – associations between lifestyle behaviours and cardiometabolic conditions

#### Physical inactivity

The proportion of people reporting no physical activity minutes increased from 23.9% in the no condition group, to 35.6% in the one cardiometabolic condition group to 49.5% in the CM group. After controlling for age and sex as confounding variables, the odds of reporting no physical activity increased with cardiometabolic disease onset. Individuals with one cardiometabolic condition and CM were 1.44 [95% CI = 1.29 to 1.62] and 2.35 [95% CI = 1.87 to 2.95] times the odds of the no condition reference group to report inactivity, respectively (Table 2). More specifically, the group with all three cardiometabolic conditions had the highest proportion of people reporting inactivity (62.0%) with an odds ratio of 4.31 [95% CI = 2.21 to 8.38]. The stroke and heart disease group and the stroke and diabetes group had 59.1 and 52.4% of people reporting inactivity with odds ratios of 2.78 [95% CI = 1.74 to 4.45] and 2.57 [95% CI = 1.44 to 4.59], respectively.

**Table 2:** Logistic regression predicting the likelihood of reporting physical inactivity

Group	Number of respondents	% physically inactive	Unadjusted Odds Ratio	Unadjusted 95% CI	Adjusted Odds Ratio^a^	Adjusted 95% CI
No cardiometabolic condition	10,159,580	23.9	1.00	reference	1.00	reference
One cardiometabolic condition	2,332,548	35.6	1.76	1.75 to 1.77^†^	1.44	1.29 to 1.62^†^
Stroke	121,326	43.7	2.47	2.44 to 2.50^†^	1.72	1.23 to 2.41^†^
Heart disease	861,064	31.7	1.48	1.47 to 1.48^†^	1.08	0.92 to 1.26
Diabetes	1,350,156	37.4	1.90	1.89 to 1.91^†^	1.69	1.46 to 1.95^†^
Cardiometabolic multimorbidity	455,712	49.5	3.12	3.10 to 3.14^†^	2.35	1.87 to 2.95^†^
Stroke and Heart disease	66,419	59.1	4.59	4.53 to 4.67^†^	2.78	1.74 to 4.45^†^
Stroke and Diabetes	49,321	52.4	3.51	3.44 to 3.57^†^	2.57	1.44 to 4.59^†^
Heart disease and Diabetes	286,515	44.4	2.55	2.52 to 2.56^†^	1.97	1.43 to 2.71^†^
Stroke, Heart disease and Diabetes	53,457	62.0	5.18	5.10 to 5.29^†^	4.31	2.21 to 8.38^†^
Total	12,947,838	26.9			–	–

^a^Controlling for age and sex; *CI* Confidence Interval; ^†^*p* < 0.05

#### Fruits and vegetables intake

Seventy-one percent of all Canadians aged 50 years and older reported eating fewer than five servings of fruits and vegetables per day. After controlling for age and sex as confounding variables, there were no significant differences in the daily consumption of fruits and vegetables by any diagnostic group when compared to the no cardiometabolic condition group (Table 3).

**Table 3:** Logistic regression predicting the likelihood of reporting eating < 5 servings of fruits and vegetables per day

Group	Number of respondents	% eating < 5 servings per day	Unadjusted Odds Ratio	Unadjusted 95% CI	Adjusted Odds Ratio^a^	Adjusted (95% CI)
No cardiometabolic condition	9,710,458	70.8	1.00	reference	1.00	reference
One cardiometabolic condition	2,134,556	72.7	1.10	1.09 to 1.10^†^	1.07	0.93 to 1.22
Stroke	88,298	72.9	1.11	1.09 to 1.13^†^	1.14	0.76 to 1.70
Heart disease	781,869	70.5	0.99	0.98 o 0.99^†^	0.94	0.77 to 1.15
Diabetes	1,264,390	74.0	1.18	1.17 to 1.18^†^	1.15	0.97 to 1.36
Cardiometabolic multimorbidity	361,056	72.9	1.11	1.10 to 1.12^†^	1.06	0.78 to 1.45
Stroke and Heart disease	43,490	75.5	1.27	1.24 to 1.30^†^	1.29	0.73 to 2.29
Stroke and Diabetes	35,852	78.2	1.48	1.44 to 1.52^†^	1.39	0.69 to 2.84
Heart disease and Diabetes	247,538	71.2	1.03	1.02 to 1.04^†^	0.96	0.64 to 1.45
Stroke, Heart disease and Diabetes	34,177	75.8	1.29	1.26 to 1.32^†^	1.30	0.61 to 2.79
Total	12,206,072	71.2			–	–

^a^Controlling for age and sex; *CI* Confidence Interval; ^†^*p* < 0.05

#### Higher stress

The proportion of people reporting higher amounts of stress increased from 18.8% in the no condition group, to 20.0% in the one cardiometabolic condition group to 22.4% in the CM group. After controlling for age and sex as confounding variables, the odds of having higher stress increased with cardiometabolic disease onset. Individuals with one cardiometabolic condition and CM were 1.41 [95% CI = 1.22 to 1.63] and 1.89 [95% CI = 1.49 to 2.41] times the odds of the no condition reference group, respectively (Table 4). More specifically, 34.0% of individuals with stroke and heart disease reported higher stress than individuals with no condition with an odds ratio of 4.50 [95% CI = 2.57 to 7.86]. Similarly, 37.7% of people with all three conditions reported higher stress with an odds ratio of 3.93 [95% CI = 2.03 to 7.61], compared to the no cardiometabolic condition group.

**Table 4:** Logistic regression predicting the likelihood of reporting higher stress

Group	Number of respondents	% higher stress	Unadjusted Odds Ratio	Unadjusted 95% CI	Adjusted Odds Ratio^a^	Adjusted 95% CI
No cardiometabolic condition	10,287,951	18.8	1.00	reference	1.00	reference
One cardiometabolic condition	2,367,663	20.0	1.08	1.07 to 1.08^†^	1.41	1.22 to 1.63^†^
Stroke	124,134	22.4	1.25	1.23 to 1.26^†^	1.90	1.26 to 2.86^†^
Heart disease	870,186	22.3	1.24	1.23 to 1.25^†^	1.83	1.49 to 2.24^†^
Diabetes	1,373,344	18.4	0.98	0.97 to 0.98^†^	1.17	0.98 to 1.41
Cardiometabolic multimorbidity	462,865	22.4	1.25	1.23 to 1.26^†^	1.89	1.49 to 2.41^†^
Stroke and Heart disease	68,994	34.0	2.24	2.19 to 2.26^†^	4.50	2.57 to 7.86^†^
Stroke and Diabetes	50,001	18.2	0.96	0.94 to 0.99^†^	1.49	0.69 to 3.22
Heart disease and Diabetes	290,975	17.5	0.92	0.91 to 0.93^†^	1.33	0.99 to 1.80
Stroke, Heart disease and Diabetes	52,895	37.7	2.62	2.56 to 2.66^†^	3.93	2.03 to 7.61^†^
Total	13,118,480	19.1			–	–

^a^Controlling for age and sex; *CI* Confidence Interval; ^†^*p* < 0.05

## Discussion

In this study, we estimated 467,749 Canadians aged ≥50 years with CM in 2016. Another 2,384,723 Canadians reported an independent diagnosis of stroke, heart disease, or diabetes and thus an elevated risk of CM. Our observations indicate an association between increasing numbers of cardiometabolic diseases and poorer lifestyle behaviours, which has important prevention and management implications. These findings are similar to Di Angelantonio et al. (2015), in their observation that risk of mortality exponentially increases with increasing numbers of cardiometabolic conditions [2].

### Prevention of CM in high-risk individuals

Our results indicate that individuals with a single cardiometabolic condition have significantly higher odds of reporting no weekly minutes of physical activity (particularly those with stroke or diabetes) and having higher stress (particularly those with stroke and heart disease) than those with no condition. These findings combined with our observation that BMI increases with increasing number of cardiometabolic conditions has important implications for the prevention of CM onset, as follows.

Both physical inactivity and chronic stress are established determinants of higher BMI [43, 44]. Higher BMI in turn has recently been shown to be strongly associated with an increased risk of CM. For example, in a large study of over 120,000 adults, the risk of developing CM ranged from double in overweight people to more than 10 times in severely obese people [26]. Therefore, when given that overweightness is associated with unhealthy lifestyles [43], along with a large body of research showing that it increases the risk of individual cardiometabolic diseases [45, 46], body composition is a plausible mechanism that may contribute to our observed associations between physical inactivity, higher stress, and CM, and warrants further investigation.

Interestingly, our findings corroborate existing evidence of a stronger relation between BMI and diabetes than with heart disease or stroke [47]. It is speculated that longer durations of being overweight are required for the development of atherosclerosis, which typically precede vascular diseases such as stroke and heart disease [47]. Our observations that BMI is highest among people with diabetes and that diabetes is the most prevalent single cardiometabolic condition among Canadians ≥50 years corroborates the hypothesized order of disease onset resulting from overweightness (i.e., diabetes first, followed by vascular diseases). Therefore, maintaining an optimal weight among individuals with diabetes is an important step towards the prevention of CM.

### Management of CM to reduce the risk of mortality

Both physical inactivity and stress are modifiable factors associated with premature mortality [48, 49]. Therefore, our finding that individuals with CM have higher odds of reporting no physical activity and having higher stress compared to those with no cardiometabolic condition contribute to an understanding of the strong association observed between CM and premature mortality [2].

Individuals with a single cardiometabolic condition report the highest levels of inactivity relative to those with other types of chronic conditions [50]. Roughly 25% of individuals with a cardiometabolic condition report no leisure time activity (e.g., walking, gardening) compared to 17% with degenerative neurological conditions, 21% with musculoskeletal conditions, and 24% with respiratory conditions [50]. Barriers to physical activity specific to individuals with cardiometabolic conditions are established, and include concerns of hypogylemia [51], fear of a heart attack [52] or stroke [53], being alone in case of a medical problem [52], and physical impairments [53]. Existing evidence suggests a positive linear association between inactivity and multimorbidity [54–56], therefore it is plausible that existing barriers specific to single conditions have additive effects that further diminish physical activity in those with CM.

Similarly, chronic conditions cause stress as a consequence of their symptoms (e.g., pain, physical limitations, decreasing independence), long-term prognosis, and other related issues such as feelings of frustration, confusion, added medical treatments and costs [57]. With multimorbidity, people face added burden of multiple appointments with an array of healthcare professionals often in different settings, as well as the need to follow complex medication regimes. Our observation of increasing reports of higher stress among people with CM compared to those without CM is therefore not surprising. Furthermore, while our findings show no change in consumption of fruits and vegetables by diagnostic group, it is concerning that 71% of our sample eat less than 5 servings of fruits and vegetables per day, and thus have unhealthy diets. This suggests a need for widespread population-based health promotion activities focusing on diet and nutrition for all Canadians aged ≥50 years regardless of cardiometabolic condition or not.

Overall, our findings indicate that chronic disease management efforts focusing on physical activity and stress among individuals with CM are necessary, and could lower mortality risk. While this is true for those with any combination of cardiometabolic condition, it is particularly relevant for stroke survivors, who report the highest rate of CM at 48.4% and the highest odds of inactivity and stress, especially if they also report having both heart disease and diabetes. Interestingly, we observe that individuals with stroke (regardless of CM or not) have lower household incomes and education levels than other diagnostic groups. When considering existing research of negative associations between income, education, and risk of chronic disease [58, 59], it is plausible that stroke survivors with low education levels are less financially secure to look after their health and disability needs, have decreased access to health services [60], and are thus at high risk for multimorbidity.

### Limitations

We were restricted to self-report data from community-living individuals. Moreover, because the CCHS excludes Indigenous people living on “Aboriginal settlement”, there is likely a non-response bias contributing to a underestimation of CM [61], especially when considering that Indigenous people a heightened risk of cardiometabolic diseases relative to non-Indigenous populations [10, 11, 62]. As well, we used a definition of CM that did not include a history of hypertension because categorizing elevated blood pressure as a binary variable would likely underestimate the effect of blood pressure on chronic disease [2]. Furthermore, the heart disease variable may include diagnoses that are not typically associated with lifestyle, such as valvular or congenital heart disease. Causality cannot be established due to the cross-sectional nature of the data thus it is uncertain if increasing numbers of cardiometabolic conditions lead to unhealthy lifestyle behaviours, as tested, or if such behaviours lead to disease, and lastly, there may be other confounding variables that we didn’t control for which may influence the results.

## Conclusion

The number of Canadians with CM or at risk of CM is high, and increasing onset of cardiometabolic conditions is associated with higher rates of physical inactivity and stress. Current models of care largely focus on individual chronic conditions. Therefore, an emerging area of research will be on identifying unique barriers to physical activity and stressors experienced by people with CM that may be modified via intervention. The development and evaluation of programs to improve physical activity in individuals with CM will likely have an additive effect at reducing stress. The study of predictors of CM is warranted in order to develop interventions targeting those that are modifiable to both prevent CM onset and better manage CM and thereby reduce their risk of mortality. Finally, priority of treatment programs should correspond with the number of cardiometabolic conditions. That is, people with more conditions should be prioritized to receive more urgent and intense management and preventive therapies.

## Data Availability

The data that support the findings of this study are available from Statistics Canada but restrictions apply to the availability of these data, which were used under license for the current study, and so are not publicly available. Data are however available from the authors upon reasonable request and with permission of Statistics Canada.

## Abbreviations

BMI: Body mass index
CCHS: Canadian Community Health Survey
CI: Confidence interval
CM: Cardiometabolic multimorbidity
SD: Standard deviation
